# Synthesis of Geopolymer from a Novel Aluminosilicate-Based Natural Soil Precursor Using Electric Oven Curing for Improved Mechanical Strength

**DOI:** 10.3390/ma15217757

**Published:** 2022-11-03

**Authors:** Muhammad Zain-ul-abdein, Furqan Ahmed, Iftikhar Ahmed Channa, Muhammad Atif Makhdoom, Raza Ali, Muhammad Ehsan, Abdullah Aamir, Ehsan Ul Haq, Muhammad Nadeem, Hafiz Zahid Shafi, Muhammad Ali Shar, Abdulaziz Alhazaa

**Affiliations:** 1 Department of Metallurgical and Materials Engineering (MME), Faculty of Chemical, Metallurgical and Polymer Engineering, University of Engineering and Technology (UET), Lahore 54890, Pakistan; 2 Department of Metallurgical Engineering, NED University of Engineering and Technology, Off University Road, Karachi 75270, Pakistan; 3 Institute of Metallurgy and Materials Engineering, University of the Punjab, Lahore 54590, Pakistan; 4 National Institute of Lasers and Optronics College, Pakistan Institute of Engineering and Applied Sciences, Nilore, Islamabad 45650, Pakistan; 5 Department of Mechanical & Energy Systems Engineering, Faculty of Engineering and Informatics, University of Bradford, Bradford BD7 1DP, UK; 6 Department of Physics and Astronomy, College of Science, King Saud University, Riyadh 11451, Saudi Arabia

**Keywords:** natural soil, sodium silicate, geopolymer, compressive strength, construction material

## Abstract

Natural soil (NS)-based geopolymers (GPs) have shown promise as environmentally friendly construction materials. The production of ordinary Portland cement is known to release significant amounts of greenhouse gas (CO_2_) into the atmosphere. The main objective of this work is to synthesize a geopolymer (GP) from an uncommon aluminosilicate-based NS and a sodium silicate (SS) activating solution that would not only minimize the emission of harmful gases, but also offer improved mechanical strength. Samples of different compositions were produced by varying the wt.% of NS from 50% to 80% and adding a balancing amount of SS solution. The drying and curing of the samples were carried out in an electric oven at specific temperatures. The degree of geopolymerization in the samples was measured by Fourier transform infrared spectroscopy, and microstructural analysis was performed using a scanning electron microscope. Mechanical tests were conducted to evaluate the range of compressive strength values of the prepared GP samples. A minimum compressive strength of 10.93 MPa at a maximum porosity of 37.56% was observed in a sample with an NS to SS ratio of 1:1; while a ratio of 3:1 led to the maximum compressive strength of 26.39 MPa and the minimum porosity of 24.60%. The maximum strength (26.39 MPa) was found to be more than the reported strength values for similar systems. Moreover, an improvement in strength by a factor of three has been observed relative to previously developed NS-based GPs. It may be inferred from the findings that for the given NS, with almost 90% aluminosilicate content, the extent of geopolymerization increases significantly with its increasing proportions, yielding better mechanical strength.

## 1. Introduction

The demand for high performing building materials in the construction industry is not likely to abate anytime soon. In addition to having good thermal and mechanical properties, these materials are expected to possess eco-friendly behavior to meet the current needs. Ordinary cement has been used as a basic construction material for many years, despite the fact that a ton of cement production releases a ton of carbon dioxide gas into the atmosphere, which leads to the greenhouse effect [1]. A new environmentally friendly construction material, called geopolymer (GP), yielding significantly lower amounts of harmful greenhouse gases during its synthesis, was introduced almost 30 years ago by Davidovits [2]. Commercially available GP cement, which only requires the addition of water and an ambient drying temperature, is generally regarded as one-part GP. A two-part GP, on the other hand, involves a combination of an aluminosilicate precursor with an alkali activator solution, such as silicates and/or hydroxides of sodium and potassium [3,4]. A geopolymerization (GPN) reaction takes place through a series of processes, viz. the dissolution of the precursor in the presence of an activator, the formation of Si–O–Si and Si–O–Al networks, the polycondensation of 3D tectosilicates, and dehydroxylation to produce a solid structure [5]. Thermal activation around the temperature range of 60–200 °C is often necessary to complete the GPN reaction. The dwell-time at these curing temperatures, however, varies from a few minutes (~5 min) to several hours (~24 h) depending upon the heat source, such as microwave radiation or an electric/gas oven [6,7]. Curing/drying is found to improve the mechanical properties, though it results in the distribution of undesirable porosity throughout the structure [8]. High levels of porosity give rise to a foamy structure, which, in turn, enhances the thermally insulative characteristics of GPs at the expense of the mechanical strength. Therefore, finding an optimal combination of low thermal conductivity and high mechanical strength is the subject of several ongoing research works, where mixing of the precursors and activators in varying compositions is often tried and tested to obtain better thermo-mechanical properties. The GPs not only have good mechanical and fire-resistant properties, but are also light weight relative to other commercial construction and building materials. Given these salient features, they can conveniently be used in civil structures in the form of bricks and blocks [9,10].

GPs have recently been synthesized using many precursors, such as fly ash [11,12], bottom ash [13,14,15], metakaolin [16,17], slag [18], glass [19,20], red mud [21], and kaolinite [22,23]. Here, the fly and bottom ashes are a more common choice for precursors; unfortunately, they carry harmful contents, including lead and cadmium [3]. Kaolinite clay and natural soil (NS) have an advantage over other precursors in that they favor relatively green processing. The former, however, involves the process of calcination and therefore, has high processing costs compared to the latter. Consequently, NS is getting more attention for the production of GP due to its ready availability, low-cost processing, and environment friendliness [24,25,26].

However, limited results have been reported in literature so far regarding the use of aluminosilicate-based soil as a raw material for GP [26,27,28,29]. These soil-based GP mortars delivered compressive strength values ranging between 7.1~22.7 MPa. Hence, one of the main objectives of this work is to improve the compressive strength of NS-based GP. In addition, the aluminosilicate content of the soils used in these works was significantly less compared to that of the NS used in the present work, e.g., Al_2_O_3_ + SiO_2_~80 wt.% in [26,27,28,29]. To compensate for this deficiency, researchers often have to add other clays that are rich in aluminosilicate content to improve the degree of GPN. The novel aluminosilicate-based NS precursor used in the current research contains Al_2_O_3_ + SiO_2_ greater than 90 wt.%, which not only eliminates the need of adding strengthening clays, but also yields relatively better mechanical strength for an optimum combination of NS and sodium silicate (SS) solution. Hence, the GP produced was stronger, as well as cheaper, than those created in similar systems.

In this study, the GPN of NS with SS solution in varying proportions was carried out, followed by high temperature curing (>200 °C). During the first phase of this investigation, GP samples were cured by means of microwave radiation, leading to very high porosity and a compressive strength of ~8 MPa [8]. With an aim to further improve the structural integrity of NS-based GP samples, the effect of electric oven heating on porosity and compressive strength has been investigated in the present work.

## 2. Materials and Methods

### 2.1. Soil Treatment

**Figure 1:**
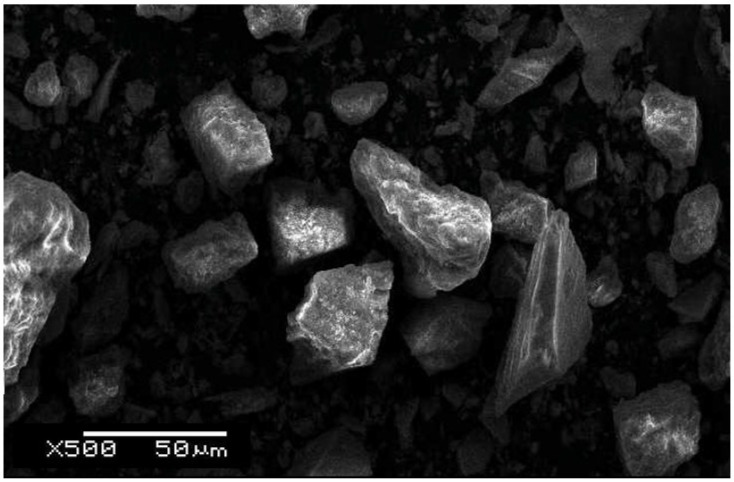
NS micrograph at 50 µm after sieving through a 75 µm sieve.

The locally available aluminosilicate NS was used as the main precursor to prepare GP samples, while SS solution was added as an activating agent. This particular type of NS, also known as alluvial soil, covers more than 40% of the land area of the Indo-Pak subcontinent [8]. NS was initially ground by rod milling with three rods of high carbon steel, operating at 220 V for 20 min. After the coarse grinding of the soil, it was sieved to a 200 mesh size (~74 µm). The un-sieved soil was ground again to obtain the desired particle size. Grinding was followed by drying in an oven at 200 °C for more than an hour to make sure that the moisture contents of the NS was completely eliminated. Since GPN is a surface reaction, the purpose of grinding was to minimize the particle size so that the surface area for the GPN could be maximized. For this reason, the ground NS was sieved through a series of standard sieves and the particle size of less than 75 µm (mesh # 200) and more than 53 µm (mesh # 270) was separated for further processing. The un-sieved soil which was coarser than 75 µm was ground again to achieve the desired size, while fine grained soil (<53 µm) was discarded. Nevertheless, even after a vigorous shaking of the sieve columns, a limited fraction of fine grains (<53 µm) was still present within the selected particle size range of 53~75 µm. This may be seen in Figure 1, where most of the NS grains are of the desired size, whereas some very fine grains are also visible. The NS composition, as determined by X-ray fluorescent spectrometry, is reported in Table 1.

### 2.2. Sample Preparation

**Figure 2:**
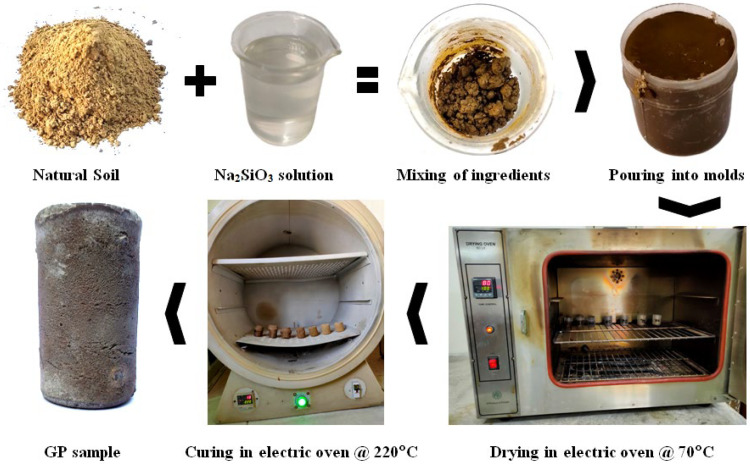
Process flow chart for the preparation of GP samples.

Two parts of powdered SS (Na_2_O.SiO_2_) were mixed with three parts of water to obtain the SS solution of desired molarity. Different compositions of NS, ranging from 50–80 wt.% with an increment of 5 wt.%, were thoroughly mixed with SS solution. Seven different mixtures were prepared and assigned codes based on the NS weight fractions. For instance, GPS50 referred to a GP sample (GPS) containing 50 wt.% NS and the remaining wt.% of SS solution. Mixing was carried out in open beakers to obtain the slurry of each composition, including GPS50, GPS55, GPS60, GPS65, GPS70, GPS75, and GPS80. The soil slurry produced was then transferred to cylindrical plastic molds (dimensions: 110 mm height × 25 mm diameter) that were not sealed from the top to allow for evaporation of water during the drying and curing processes. The molds were placed in an electric oven (PCSIR, 60 Lit.) for an initial drying at 70 °C for 24 h. The samples were then oven-cooled and removed from the molds before again being placed in the oven for curing and GPN. During curing, a temperature of 220 °C was maintained for a period of 2 h. This is the most critical step, since GPN reaction takes place at these high temperatures [6,7]. Finally, the GP samples were oven-cooled once again and retrieved for testing. The process route of the sample preparation is summarized in Figure 2.

### 2.3. Characterization Methods

The chemical composition of NS was determined with the help of an X-ray fluorescence spectrometer (XRF, Baltic Scientific Instruments, Riga, Latvia). The bulk and true densities of the GP samples were calculated as per the standards BSEN12390-7, B212-17, and D854-06 using a pycnometer (Gay-Lussac, 50 mL, Cole-Parmer, IL, USA). The porosity percentage was calculated using Equation (1), where the bulk density refers to the density of the whole GP specimen, including air voids/porosity, while the true particle density implies the density of only the solid particles, excluding porosity.


(1) $$\mathrm{Porosity}\;(\%)\;=\;[1-(\;\frac{\mathrm{Bulk}\;\mathrm{density}}{\mathrm{True}\;\mathrm{particle}\;\mathrm{density}})]\times 100$$

Compression tests on four samples of each composition were carried out on a universal testing machine TIRAtest2810 (Schalkau, Germany) equipped with a 10 kN load cell, according to the ASTM C109 standard. The machine was operated in displacement mode, and the crosshead speed was maintained at 10 mm/min. Sample dimensions, i.e., height and diameter, were measured before the testing using a digital vernier caliper with a least count of 0.01 mm. Note that the specimens were ground to maintain an average diameter of 25 mm and a height of 50 mm.

Fourier transform infrared (FTIR) spectroscopy was performed on a PerkinElmer-ATR instrument to evaluate the extent of GPN. The samples were scanned from a 400 to 4000 cm^−1^ wavenumber at a scanning rate of 2 mm/s, with a resolution of 4 cm^−1^. The surface morphology of the samples and the porosity distribution were examined under a field emission scanning electron microscope (FE-SEM) TESCAN MAIA3 microscope, equipped with an Octane Elite EDAX detector (Brno-Kohoutovice, Czech Republic). Here, scanning was done with a secondary electron detector under high vacuum at 20 kV acceleration voltage, without any sample preparation, i.e., no sputter coating on any sample. Each sample was cut into a small piece of an approximate area of 1 × 1 mm^2^, and at least 3 images of each sample were taken at the prescribed magnifications.

## 3. Results and Discussion

### 3.1. Density and Porosity Evaluation

The density and porosity values corresponding to each sample are reported in Table 2 and compared with the initial solid/water ratio of the sample. It may be observed that solid/water ratio and the overall bulk density increase with increasing NS contents, where the maximum and the minimum density values were found to be 1.857 g/cm^3^ and 1.348 g/cm^3^ for GPS75 and GPS50, respectively. On the contrary, the porosity percentage decreases with increasing NS.

Recall that the NS was almost completely dried before mixing in the SS solution, which implies that the SS solution was the only source of water in the slurry. For this very reason, the evaporation of water during the drying and curing processes and hence, the porosity contents, were greater in the samples with high SS solution content. It should also be mentioned that the maximum porosity (37.56%) for GPS50 in the present work was lower than the minimum porosity (37.82%) observed previously for a similar system [8] when microwave radiation was used for the curing purpose.

**Figure 3:**
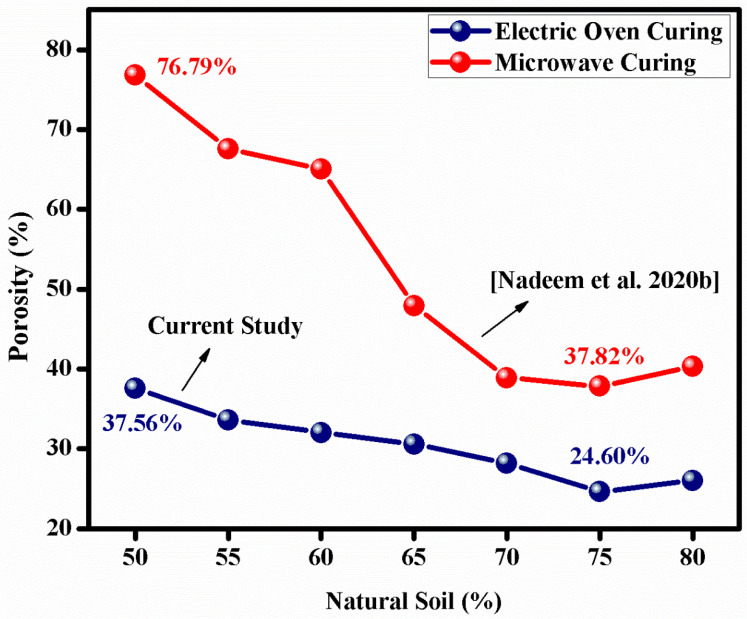
Percentage of porosity as a function of NS weight fraction—comparison of oven heating vs. microwave curing [8].

Figure 3 compares the porosity level as a function of the NS contents for both the studies. It must also be noticed that the curing temperature in both the investigations was same, i.e., 220 °C, whereas the dwell-time in the case of microwave curing [8] and electric oven curing (present work) were 4 min and 2 h, respectively. This means that the microwave radiation brings about a rather vigorous GPN reaction in a very short span of time, since it has the tendency to quickly penetrate through the plastic mold, interact directly with the constituents of the sample, and initiate GPN at once throughout the specimen. Owing to a sharp increase in temperature, the water vapors combine to form relatively larger pores that almost ‘burst’ through the top of the sample to eventually result in a so-called ‘foamy’ structure. Electric oven heating, on the other hand, gradually heats the sample outside-in. As the heating is slow, so is the curing of the sample, as well as the evaporation of the water. The complete evaporation of the water, therefore, requires several hours. The porosity produced in this way is generally small in size and evenly distributed, as compared to that formed in microwave cured sample; hence, this method is unable to yield a foam-like structure.

In a similar soil-based GP, Zaidi et al. [26] reported a maximum porosity of 63.1% with a bulk density of 0.863 g/cm^3^ for a soil-to-alkali ratio of 7:3, which closely resembles the GPS70 (Table 2) of the present work with respect to composition. However, the bulk density of the latter (GPS70~1.734 g/cm^3^) is twice that of the former (~0.863 g/cm^3^), which, in turn, led to the porosity level of the latter (GPS70~28.19%) being two times less than the former (63.1%). Clearly, the GP produced by Zaidi et al. [26] exhibited less mechanical strength, but showed better insulation properties. Although Uddin et al. [29] succeeded in achieving the porosity level in a red soil-based GP to as low as 21.4%, for an alkali-to-solid ratio of 0.4, equivalent to solid:alkali = 71:29, the maximum compressive strength of GP mortar remained less than 8 MPa. Note that the maximum strength (26.39 MPa) found in the current investigation is at least 3 times more than that of the GP developed by Uddin et al. [29]. Further comparisons of strength values with those in the published literature will be presented in the Section 3.5.

### 3.2. Fourier Transform Infrared Spectroscopy

**Figure 4:**
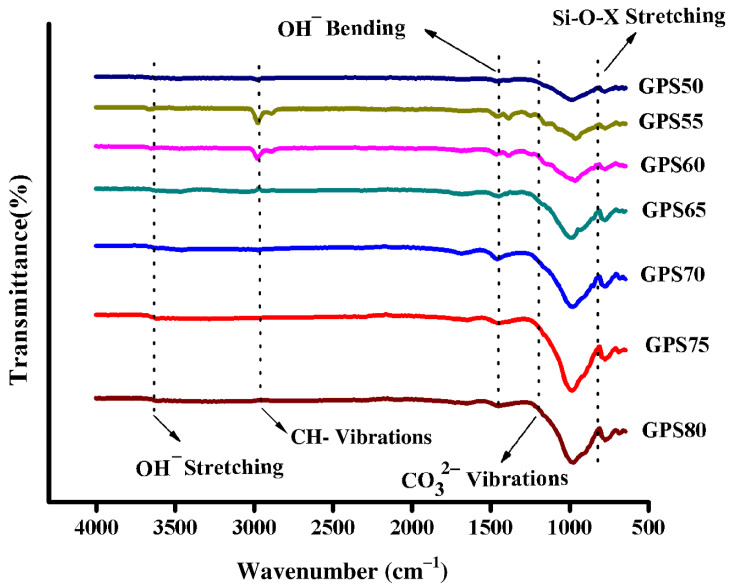
FTIR spectra of GP samples.

Before the discussion on the mechanical strength is developed, it is important to evaluate the degree of GPN in each sample. Figure 4 summarizes the results of FTIR spectroscopy which were used to quantify the extent of the GPN reaction. The FTIR spectra indicate -OH bending vibrations at 1451 cm^−1^, while the absorption bands at 3627 cm^−1^ exhibit -OH stretching vibrations due to the presence of water within the SS solution. Similarly, the peak at 2960 cm^−1^, more pronounced for GPS55 and GPS60, may be attributed to the presence of CH–bonds due to organic contamination of the NS. A relatively shallow band between 1003 cm^−1^ and 820 cm^−1^ for all the samples mainly identifies the asymmetric vibrations of the Si-O-X (X = Al or Si) network, which is a typical indicator of the GPN [8,30,31,32]. Likewise, the peak at 1208 cm^−1^ shows stretching vibrations of CO_3_^2−^ [26] that appeared due to the carbonate (Na_2_CO_3_) formation from the Na^+^ in SS solution and environmental CO_2_ during the mixing of the GP ingredients.

Note that the GPN or Si-O-X band (1003–820 cm^−1^) grows increasingly shallower from GPS50 to GPS80, wherein NS increases from 50 to 80 wt.%. Since the degree of GPN depends upon the area under this region of the curve, it may be inferred that the GPN is proportional to an increase in NS and a decrease in SS contents. Nevertheless, a critical amount of SS solution corresponding to NS contents is always required to achieve maximum GPN. This is because too little SS solution would be unable to completely ‘wet’ the NS particles, while an excess amount would lack sufficient aluminosilicate precursor to continue the GPN reaction.

**Figure 5:**
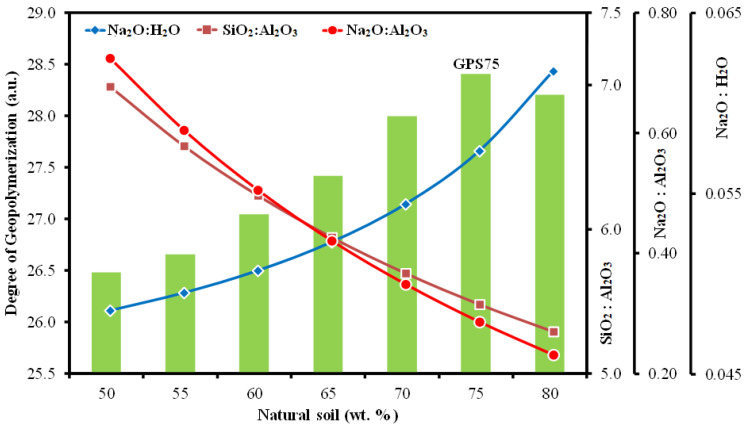
Degree of GPN vs. molar ratios of SiO_2_:Al_2_O_3_, Na_2_O:Al_2_O_3_, and Na_2_O:H_2_O as a function of NS wt. fractions.

This can be understood with the help of Figure 5, where the degree of GPN of all the samples is compared with each other by calculating the area under the Si-O-X band. Note that the maximum degree of GPN was found in the GPS75 and not the GPS80, which clearly indicates that for a given composition of NS, 20 wt.% of SS solution is insufficient to wet all the NS particles and, hence, incapable of delivering maximum GPN. Similarly, the samples with excess (>25 wt.%) SS solution show a significant decrease in GPN, as not enough NS is present in the sample to continue the reaction.

Given that a large variety of precursors, such as metakaolin, fly and bottom ashes, slags, etc., are being used worldwide for the synthesis of GPs, researchers [33,34,35,36,37] have proposed a rather quantifiable way of identifying better performing GPs in terms of the molar ratios of their major constituents, which include alumina, silica, sodium oxide, and water. In a comprehensive review, Khale and Chaudhary [38] summarized that for superior mechanical properties, a kaolin, metakaolin or fly ash based GP must contain SiO_2_:Al_2_O_3_~3.3–4.5, M_2_O:SiO_2_~0.2–0.48, M_2_O:Al_2_O_3_~0.8–1.6, and M_2_O:H_2_O~0.04–0.1, where M can either be Na or K. Since the chemical composition of NS (see Table 1) in the present work is different from that of commonly available fly ash or metakaolin, it is expected that the molar ratios of the major constituents will not strictly follow the above-mentioned limits. However, certain parallels can still be drawn. For instance, in addition to the degree of GPN, Figure 5 also illustrates the evolution of molar ratios of Na_2_O:H_2_O, SiO_2_:Al_2_O_3_, and Na_2_O:Al_2_O_3_. Note that Na_2_O:H_2_O lies between 0.047–0.061, which is well within the above reported range. However, the molar ratios SiO_2_:Al_2_O_3_ and Na_2_O:Al_2_O_3_ vary between 5.3–7.0 and 0.24–0.73, respectively, which, although not in complete agreement, are still close to lower limit of the prescribed ranges. Finally, it should also be noted that higher values of these molar ratios do not guarantee a higher degree of GPN. GPN, in fact, depends strongly upon several other factors, including aluminosilicate content, particle size, wetting characteristics, curing temperature, dwell-time, heat source, etc. In this work, the sample GPS75 showed the maximum degree of GPN and the molar ratios Na_2_O:H_2_O, SiO_2_:Al_2_O_3_, and Na_2_O:Al_2_O_3_ of 0.057, 5.49, and 0.29, respectively.

### 3.3. Scanning Electron Microscopy

**Figure 6:**
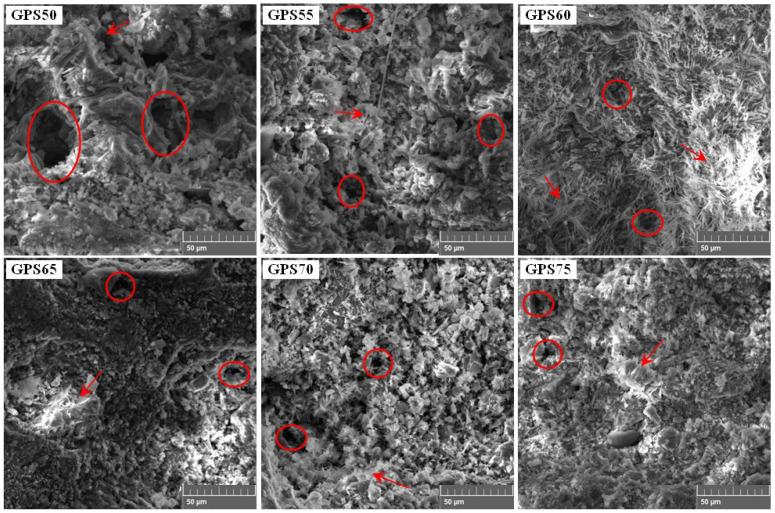
SEM images of GPS50, GPS55, GPS60, GPS65, GPS70, and GPS75 (Circles show porosity, arrows indicate NS agglomerates).

SEM analysis revealed critical information regarding the surface morphology of the GP samples and the porosity size and distribution. Figure 6 illustrates SEM images of all the samples except that of GPS80. The encircled regions in the micrographs indicate the existence of porosity in the samples, while the arrows point to the agglomerates/lumps of reacted NS. Note that from GPS50 to GPS75, as the percentage of NS increases, the porosity size decreases from very coarse (~20 μm) to very fine (~2 μm). Furthermore, the GPS50, on one hand, demonstrates random porosity distribution and large lumps of reacted soil, while on the other hand, the GPS75 presents a relatively uniform distribution of porosity, with smaller regions of reacted soil. Clearly, the presence of coarse porosity in GPS50 can only be attributed to the excess amount of water in the SS solution, as all the processing parameters for the whole batch of samples were constant.

**Figure 7:**
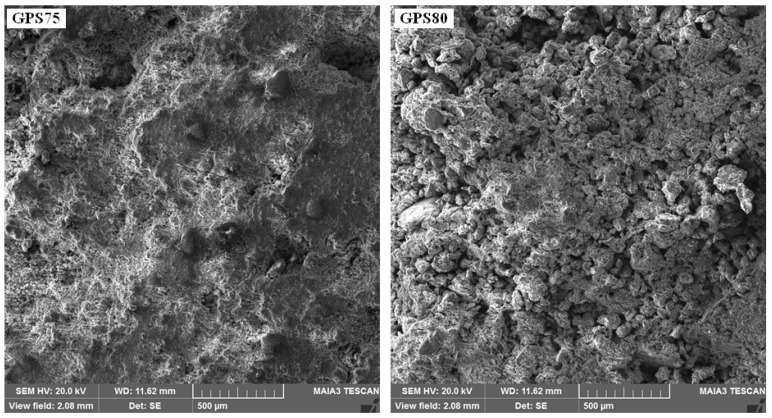
SEM images of GPS75 and GPS80 showing a continuous and agglomerated matrix, respectively.

When the amount of SS solution is lower, as in GPS75 and GPS80 (see Figure 7), the water contents are barely enough to form a fluid or paste-like slurry and are just sufficient to ‘wet’ the NS by developing a thin layer around its particles. During curing in the electric oven, heating gradually takes place from the surface of the sample to the center, thereby evaporating the thin layer of water immediately and thoroughly bringing about the GPN reaction at the interface of the NS particles. Since the water content here are not enough to agglomerate and form larger pores, fine porosity appears throughout the specimen. Moreover, the degree of GPN increases due to the NS and SS reaction taking place at a larger surface area. However, if the SS solution is less than required, as in GPS80, the GPN is unable to reach the maximum value. This effect may also be observed in Figure 7, where the GPS75 with a critical amount of SS solution and maximum GPN exhibits a relatively continuous matrix, while the GPS80 with a lower amount of SS solution reveals a rather discontinuous matrix, with small agglomerates of NS particles, which is also likely to increase the porosity fraction of the sample.

### 3.4. Mechanical Strength Analysis

**Figure 8:**
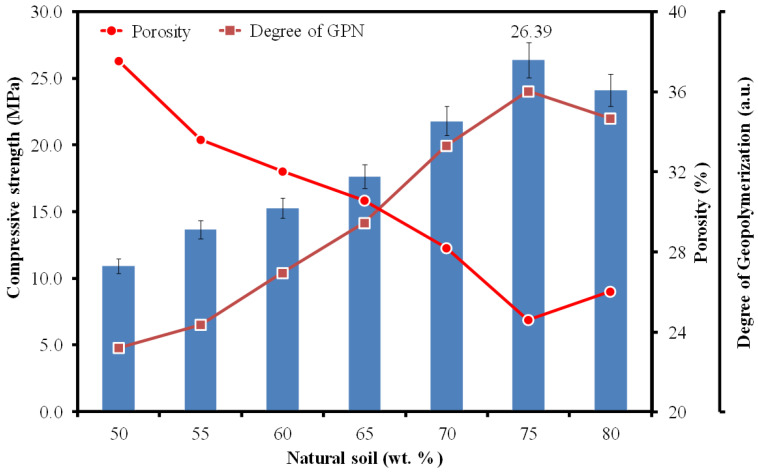
Compressive strength vs. porosity and degree of GPN as a function of NS wt. fractions.

The compressive strength values of the GP samples range from 10.93 MPa to 26.39 MPa, as reported in Table 2. Figure 8 draws a comparison between the strength, porosity, and degree of GPN as a function of the NS weight fraction. Note that the compressive strength is directly proportional to the GPN, while inversely proportional to the porosity.

It has been discussed earlier that the maximum degree of GPN is achievable only through an optimal combination of NS and SS solution, which in the present case, applies exclusively to GPS75. It may now be noticed that the same sample reached the maximum compressive strength. Since the GPN reaction takes place only on the surface of the NS particles, modifying their texture [7], the formation of a strong Si-O-X network is completely achieved by a limited amount of SS solution present in the system. The excess amount of SS not only develops an Si-O-X network, but also leads to the formation of a dendritic needle-like weak structure [8] that has the tendency to fail under compressive loading before transferring the stresses to the Si-O-X network. This is the very reason for the decrease in compressive strength with a decreasing NS to SS ratio. In fact, it would be convenient to state that increasing the NS:SS from 1 to 3 increases the strength by a factor of more than 2. It is, however, difficult to isolate the role of the porosity fraction from those belonging to its size and distribution. For the moment, the porosity curve merely indicates that with its maximum volume fraction, the strength drops to the minimum, and vice versa. These findings are consistent with the observations made previously in similar works [3,26].

### 3.5. Comparative Analysis with Published Works

**Figure 9:**
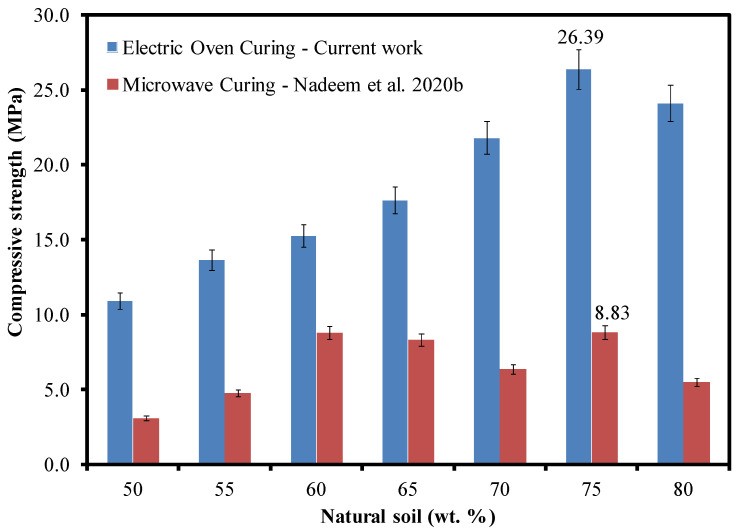
Comparison of compressive strengths obtained through electric oven and microwave radiation curing as a function of NS wt. fractions [8].

It is of interest to correlate the compressive strengths of the GP samples with the results of previously published work [8]. Figure 9 compares the strength values of electric oven-cured GP samples (present work) with those of microwave radiation-cured [8] samples as a function of the NS weight fraction. It may be observed that the compressive strengths of all the former samples are greater than those of the latter. Nevertheless, both the datasets yielded the maximum strength for the samples of the same composition, i.e., GPS75. This ensures that the NS:SS ratio does play a significant role in defining the mechanical properties of these GPs. As mentioned earlier, curing via microwaves, although taking only 4 min, brings about an intense GPN reaction that renders the samples highly porous (see Figure 3). Consequently, this produces a relatively weaker structure. On the contrary, a slowly progressing GPN reaction over a period of 2 h, as in case of electric oven heating, ensures the gradual drying and evaporation of water from the external layers first before reaching the center, thereby leading to the formation of a less porous structure. Hence, the resultant GP structure possesses more strength than that obtained by microwave curing. Note that the maximum compressive strength of the electric oven-cured sample (~26.39 MPa) is almost 3 times more than that of the microwave-cured sample (~8.83 MPa). This highlights not only the importance of the curing process, but also the possibility of tailoring the strength values according to the application. Although an optimum composition for better mechanical properties is being proposed, a new cost-based optimum must be sought out by the designer, keeping in view the operational costs of microwave and electric ovens for a duration ranging from a few minutes to several hours.

Table 3 summarizes and compares the results of the compressive strength of different soil-based GPs. The comparison illustrates that the strength of the NS-based GP prepared in the current investigation is better than that of several similar systems studied previously. Normalizing the strength values with respect to the ordinary cement mortar indicates an improvement in strength of more than ten times that of NS-based GPs produced in recent years. The aluminosilicate NS precursor, the optimum combination with SS solution, the curing conditions, and the synthesis parameters eventually led to the formation of a GP that has the strength comparable (~94%) to that of ordinary cement mortar. This shows the potential for the use of NS-based GPs, mainly in construction applications where strength is the main concern. It should also be mentioned that although increasing the porosity fraction decreases the mechanical strength (see Table 2), such GPs can be successfully exploited in thermal management applications where insulation and heat preservation is highly desirable.

## 4. Conclusions

With the objective of improving their compressive strength, NS-based GPs with varying contents of an activating agent have been synthesized in this work. Based on the above discussion, the following conclusions may be drawn.

- A high aluminosilicate content in NS is critical to achieving high-strength GPs.
- The optimization of the soil-to-alkali or solid-to-water ratio is crucial to maximize the degree of GPN.
- Curing in a microwave or an electric oven significantly influences the porosity and strength levels of the prepared GPs.
- High-strength GPs are desirable to improve structural integrity, while highly porous GPs may be exploited as thermal insulators.

## Figures and Tables

**Table 1:** Natural soil composition [8].

Components	Percentage (%) by Mass
SiO_2_	62.81
Al_2_O_3_	22.37
Fe(OH)_3_	11.47
TiO_2_	1.11
Calcite	0.92
Na_2_O	0.81
Other impurities	0.51

**Table 2:** Physical and mechanical properties of GP samples.

Sample ID	Solid/Water Ratio	Bulk Density (g/cm^3^)	True Particle Density (g/cm^3^)	Total Porosity (%)	Compressive Strength (MPa)
*GPS50	2.27	1.348	2.159	37.56	10.93
GPS55	2.64	1.538	2.317	33.62	13.67
GPS60	3.09	1.589	2.338	32.04	15.27
GPS65	3.68	1.648	2.374	30.58	17.65
GPS70	4.46	1.734	2.415	28.19	21.82
GPS75	5.55	1.857	2.463	24.60	26.39
GPS80	7.19	1.811	2.448	26.02	24.13

*GPS50: 50 wt.% NS + rest SS solution (applies to all sample IDs).

**Table 3:** Compressive strength comparison of NS-based GP with those in published works.

GP Mortar Material	Compressive Strength (MPa)	Normalized Strength	Reference
Natural soil	2.41	0.09	[26]
Red soil	7.1	0.25	[28]
Natural soil	8.83	0.32	[8]
Granite powder	22.0	0.79	[24]
Loam natural soil	22.7	0.81	[27]
Natural soil	26.39	0.94	Present work
Ordinary cement mortar (*not a GP*)	28.0	1.0	[39]

## Data Availability

Not applicable.
